# The Importance of Sleep and Circadian Rhythms for Vaccination Success and Susceptibility to Viral Infections

**DOI:** 10.3390/clockssleep4010008

**Published:** 2022-02-16

**Authors:** Nina C. M. Schmitz, Ysbrand D. van der Werf, Heidi M. Lammers-van der Holst

**Affiliations:** 1 Department of Anatomy and Neurosciences, Amsterdam UMC, Vrije Universiteit Amsterdam, Amsterdam Neuroscience, De Boelelaan 1117, 1081 HV Amsterdam, The Netherlands; ninischmitz@gmail.com (N.C.M.S.); yd.vanderwerf@amsterdamumc.nl (Y.D.v.d.W.); 2 Department of Public Health, Erasmus University Medical Center, Doctor Molewaterplein 40, 3015 GD Rotterdam, The Netherlands

**Keywords:** sleep, circadian rhythms, COVID-19, coronavirus, SARS-CoV-2, cold, immune function, vaccination

## Abstract

Sleep and circadian rhythms are closely involved in the immune system and its regulation. Here, we describe this relationship and provide recommendations regarding the influence of sleep and circadian rhythms on vaccination success. We review studies investigating how viral susceptibility is influenced by changes in immunological parameters as a consequence of sleep deprivation. Short sleep duration and poor sleep efficiency both appear to be strong factors leading to greater vulnerability. In addition, both sleep duration and the time of day of the vaccination seem to be associated with the magnitude of the antibody response after vaccination. Based on these findings, a recommendation would consist of a sleep duration of 7 h or more every night to both reduce the risk of infection and to optimize the efficacy of vaccination with respect to circadian timing. Improving sleep quality and its circadian timing can potentially play a role in preventing infection and in vaccination benefits. In conclusion, sufficient (or longer) sleep duration is important in both reducing susceptibility to infection and increasing antibody response after vaccination.

## 1. Introduction

Sleep is an essential part of daily life, and, over the last few years of research, it has become increasingly evident that sleep is a key component for many vital functions. It has an important role in, among others, the modulation of immune responses, cognition, and disease [1]. Adult humans naturally sleep in one consolidated bout during the nighttime hours and remain awake for an extended period throughout the day. This ability to remain awake or asleep for an extended time is due to a complex interaction of the circadian timing system and the homeostatic sleep–wake system [2]. The circadian process contributes a rhythmic variation in sleep–wake propensity and promotes wakefulness during the day and sleep at night. The sleep homeostatic process describes a build-up of sleep pressure with time awake and the dissipation of that sleep pressure during sleep [2,3]. Sleep is longest and least disturbed when the sleep episode occurs at the biological time the circadian system most strongly promotes sleep [4,5]. 

Our circadian system imposes a near 24-h rhythmicity on behavior, physiology, and metabolism. The suprachiasmatic nucleus (SCN) in the hypothalamus is the ‘master clock’ that generates and regulates the body’s circadian rhythms and helps to synchronize them to the environmental 24-h light–dark cycle [6]. Next to this master clock, peripheral clocks are present in organs and tissues such as the spleen, white and brown adipose tissue, pancreas, and lymph nodes, where they coordinate cellular processes. Circadian rhythms are found in specific cells of our immune system, e.g., macrophages, monocytes, neutrophils, and NK cells [7]. Sleep and circadian rhythms are interconnected, and research has shown that they are important contributors to immunological functions. 

The immune system of our body fights off infections and repairs cellular damage through inflammatory processes. The localization of an inflammatory response is dependent upon chemical signals known as cytokines. An acute response consists of localized inflammatory activity with a short duration. Chronic inflammation is more systemic, with constant elevated levels of circulating cytokines [8]. The immune system can be divided into two subsystems: the innate and the adaptive immune system. The innate immune system is the first line of defense against invading pathogens and is considered as the “nonspecific” system. The innate immune system comprises skin, all mucosal membranes, natural killer T-cells (NK), and phagocytic cells (monocytes, neutrophils, eosinophils, macrophages, and basophiles). The adaptive immune system is more specialized, taking more time to respond, but is capable of specifically targeting a pathogen entering the body. Taken together, all these components are critical to host resistance to infectious diseases, such as common cold and severe acute respiratory syndrome coronavirus-2 (SARS-CoV-2) infection. Another hallmark of the adaptive immune system is the ability to “remember” the pathogen for a faster response the next time the same pathogen is encountered. This immunological memory system is also active throughout the production of antibodies after vaccination. The system consists of T lymphocytes, B lymphocytes, and antibodies circulating in blood and other body fluids. 

In our modern society, sleep problems and disruptions to normal circadian regulation are common. These disruptions include sleep deprivation, social jet lag, and rotating shift work. Circadian disruptions are associated with poorer health, as can be seen, for example, in shift workers who constitute a vulnerable group facing sleep loss and circadian misalignments in relation to negative health outcomes [9,10]. A commonality shared by these adverse health outcomes is improper inflammatory response. Chronic exposure to circadian and sleep disruptions may systemically elevate circulating cytokines, potentially leading to adverse inflammatory-related diseases. 

Previous work has provided evidence showing that sleep and circadian alignment boost the body’s immune response. In a US national representative sample of adults, Prather 2021 showed that both short and long sleep duration, as well as a rotating shift works schedule, were significantly associated with increased rates of experiencing a head or chest cold. Compared to adults who slept 7 to 8 h, participants who reported sleeping 9 h or longer were 20% more likely to report a head or chest cold [11]. This linkage to long sleep duration was also found by Patel and colleagues (2012), where prolonged sleep was linked to pneumonia [12]. These studies are consistent with other U-shaped associations between sleep duration and health outcomes. 

Another important aspect is that the amount of sleep a person had at the time of vaccination against viral infections has an influence on their immune response. A good night’s sleep can positively influence the immune response such that the vaccination efficacy is higher. Both aspects, immune function and vaccination efficacy, have been studied extensively over the last decade. The precise effects of sleep and circadian rhythms need to be elucidated further, however, in order to gain a better idea of the importance for both aspects [13,14,15]. This review goes into greater depths regarding the role of sleep and circadian processes in susceptibility to viral infections and vaccination success (i.e., effective immunological response to vaccination). Moreover, we highlight how these immunological mechanisms are related to the global ongoing SARS-CoV-2 pandemic, give an overview of the latest studies in this area, and provide recommendations for research and healthcare. 

## 2. Effect of Circadian Rhythms and Sleep on Viral Susceptibility

It is important to understand the relationship between sleep, circadian rhythms, and the immune system. It is well known that there is a bidirectional link between the immune system on one side and sleep and circadian rhythms on the other side. Many immune cells and immune responses are regulated in a time of day-dependent manner, e.g., concentrations of cytokines can fluctuate throughout the day [16]. However, it is also thought that cytokines influence sleep mechanisms and patterns when immune responses are active [17]. The focus of this review is on the direction in which sleep and the circadian system affects immune system function. It is thought that a lack of sleep as well as circadian disruption are associated with an increased susceptibility to viral infections. 

### 2.1. Circadian Rhythms and the Immune System

Recent findings have shown a critical role for circadian rhythms in immune functioning. This is because both the innate and adaptive immune system are regulated in a time of day-dependent manner. Circulating numbers of lymphocytes fluctuate throughout the day in healthy humans. To date, numerous other immune cells and other key players such as cytokines have been found to adapt to the circadian rhythm as well [18]. Genetic studies in human cells and mice have shown at least three types of immunological activity under the influence of the circadian rhythm. First, there is the regulation of cytokine secretion. Cytokines are important mediators of immune responses. The key components of the circadian clock are the so-called “clock genes”, which are either transcription factors or transcription factor regulators. Those clock genes are capable of directly activating or repressing gene expression of certain cytokines. During normal sleep, the secretion of cytokines favors a more proinflammatory state, which is reduced during the daytime. Disruption of clock function causes a shift in macrophages, i.e., the macrophages will secrete high amounts of cytokines. Second, the circadian clock regulates the trafficking of myeloid and lymphocyte subsets. For example, this trafficking includes removal of leukocytes from the blood and organs to the lymph nodes during sleep, allowing for proinflammatory processes. This process is complex, and the circadian regulation is important in optimizing this immune activity. Third, the differentiation and maturation of leukocyte subsets that are of clinical importance appear related to the circadian clock. This connection, however, is among the least studied. The circadian clock controls both the innate and adaptive immune systems, thereby preventing a state of constant immune activation. Gibbs and colleagues (2014) showed that anti-bacterial immune responses, during inflammatory lung diseases, are under the control of the circadian rhythm. The clock genes activate the expression of a certain chemokine ligand (CXC-chemokine ligand 5) that regulates the recruitment of neutrophils to the lungs, starting the immune response [19]. In sum, under normal circumstances, sleep and the circadian rhythm ensure an anti-inflammatory state during the daytime and a proinflammatory state during nighttime. 

### 2.2. Sleep and Immune Function

It is thought that the clock may exert its influence on immunity by regulating sleep, and, given the interdependence of sleep and circadian rhythms, it is not surprising that immunological activities affected by insufficient sleep resemble those that are affected by circadian disruption. Studies investigating the effects of induced sleep deprivation and short habitual sleep (i.e., 6 h or less sleep per night) found that this resulted in a higher proinflammatory cytokine secretion. In addition, the number of circulating immune cells, such as neutrophils and B-cells, was higher. This suggests that sleep plays a role in leukocyte trafficking similar to the effect of the circadian clock [16]. Inflammation is important for the organism’s survival during disease, since it is part of the immune response against invading pathogens. A chronic imbalance in this inflammation process, however, has negative effects on the immune response. Should a constant proinflammatory state be present, a breakdown of immune tolerance may follow. Alterations in cellular physiology can lead to impairments in the normal immune function. This leaves an organism more vulnerable to diseases such as viral infections. 

There are many studies that have investigated the effects of sleep manipulation in the form of sleep deprivation on various immune parameters. Primarily, immune parameters such as the number of leukocytes, subsets of leukocytes, cytokine levels, complement factors in the blood, as well as cell cytotoxicity, have been investigated. Looking at leukocyte count and distribution, it was found that adequate sleep reduced these numbers when compared to total and partial sleep deprivation [20,21], but note that other studies were not able to detect these reducing effects [22]. Reductions in the total number of lymphocytes, monocytes, and NK cells with adequate sleep have similarly been observed [20,23]. Leukocytes are immune cells that have a strong migratory capacity and travel throughout the entire body using the blood and the lymphatic system as transport routes. The question remaining is: what happens to these leukocytes if numbers in the blood drop? It is unlikely that sleep has an impact on the proliferation of these leukocytes, since the effects are already seen within 3 h of sleep and proliferation takes longer to become evident. Therefore, it is most likely that these cells are redistributed to lymph nodes and other lymphoid organs and tissues, although this remains inconclusive [13]. It would explain why people who sleep well at night are less susceptible to any viral infection. In the lymph nodes and lymphoid organs, exposure to virally infected cells occur. If leukocytes are present here, they are able to quickly elicit an immune response. People who experience total or partial sleep deprivation have fewer of these leukocytes in their lymph nodes and more in the blood circulation, making them more susceptible to infectious disease. Cytokine levels are also influenced by sleep as a function of the circadian rhythm. There is a delicate balance between the production of proinflammatory and anti-inflammatory cytokines. In general, it seems that sleep favors a proinflammatory environment. Since this is regulated by a homeostatic response, eventually, this effect reverses and an anti-inflammatory environment arises. Studies in rats have looked at sleep deprivation and in particular REM sleep deprivation. They found increased levels of IL-1α, IL-1β, IL-6, IL-10, TNF, and IL-17A, which are inflammatory cytokines. More studies on sleep deprivation showed that sleep loss is associated with increased inflammatory response markers, such as C-reactive protein (CRP) and IL-6 [24,25]. When presented with an inflammatory challenge, the number of circulating monocytes increases, as well as the number of cytokines produced per cell, leading to a substantial increase in cytokines such as IL-6 and TNF-alpha [23,26]. This suggests that sleep deprivation and REM sleep deprivation alter immune system components, thereby influencing cytokine levels and cytokine production. This may lead to a chronic imbalance in immune function in favor of an inflammatory state, increasing susceptibility to viral infections [17]. Another aspect that has been studied regarding sleep deprivation is the activity and proliferation of immune cells. The morning after total or partial sleep deprivation, NK cell activity is reduced in humans [27]. Interestingly, an increase in NK cell activity occurs in the evening following two days of no sleep [28]. These differences could be due to the time in which the activity was measured (morning vs. evening). Another explanation could be that, without sleep, NK cell functioning may deteriorate; however, if sleep deprivation is prolonged, this NK cell activity will adapt to the situation. This will lead to an increase in NK cell activity back to normal, rather than a further decline in function. When looking at proliferation, particularly of lymphocytes, there is a similarly complex effect of sleep. On the morning after total or partial sleep deprivation, there was a reduced proliferation rate in these cells. Again, after two nights of recovery sleep, there was no change detected, and, after several nights, an increase was seen [26,28]. It is suggested that, for proliferation processes, some kind of compensating mechanism occurs after a few nights with total or partial sleep deprivation. Further research is necessary to better understand these compensatory mechanisms after sleep loss [13]. 

To conclude, sleep and circadian rhythms are becoming more recognized as crucial factors in immune functioning and how this relates to greater susceptibility to viral infections after (partial) sleep deprivation. Factors that are influenced by sleep and the circadian rhythm are cytokine levels and production; leukocyte subset numbers and distribution; and the proliferation and activity of immune cells. In addition, under normal circumstances, sleep and the circadian rhythm ensure an anti-inflammatory state during the daytime and a proinflammatory state during nighttime [18]. However, when both processes are disrupted, it results in a constant state of inflammation. Subsequently, this will make organisms more susceptible to disease, including viral infections [16]. Below, we discuss these concepts in the context of the common cold and SARS-CoV-2 virus (COVID-19) infection. 

### 2.3. Common Cold

The common cold is an infection every person deals with in their life and recurs every year, often during the fall and winter seasons. The common cold is caused by the rhinovirus. Rhinoviruses are a large family of viruses that cause many different diseases. The virus that causes the common cold often mutates, which is why an individual may be infected numerous times. While sleep deprivation has been linked to a decrease in immune function in experimental studies, there are only a few studies that have investigated the influence of sleep on the susceptibility to the common cold. The first example is a study by Cohen et al. (2009), in which sleep duration and efficiency were examined in relation with cold susceptibility. One hundred and fifty-three healthy volunteers, men and women, reported their sleep duration and efficiency over 14 days; after this, the participants were quarantined in the lab and received nasal drops containing a rhinovirus. Subsequently, the participants were monitored for the development of a clinical cold 5 days after exposure. They found an association with sleep duration as well as sleep efficiency. Less than 7 h of sleep resulted in a 2.94 higher chance of developing a cold compared to 8 or more hours of sleep (95% confidence interval [CI], 1.18–7.30). For sleep efficiency, participants were 5.50 times more likely to develop a cold when having a sleep efficiency of less than 92%. This was compared to participants with an efficiency of 98% or more (95% CI, 2.08–14.48). However, whether the participants felt rested or not was not associated with a higher susceptibility to the common cold. It turned out that both associations remained substantial after controlling for age, body mass, and health habits (smoking, alcohol use, and physical activity). However, this study showed that sleep efficiency is the stronger overall correlate of illness [29]. Prather and colleagues (2015) investigated whether sleep could predict cold incidence after experimental viral exposure. Actigraphy, in combination with sleep diaries, was used to measure sleep objectively and subjectively over a 7-day period in 164 healthy volunteers. It was found that shorter sleep duration was related to an increased susceptibility for a clinical cold. This was specifically for people who slept less than 5 h (odds ratio [OR] = 4.50, 95% confidence interval [CI], 1.08–18.69) or between 5 and 6 h (OR = 4.24, 95% CI, 1.08–16.71) compared to people who slept more than 7 h per night. Again, this association was independent of common covariates, such as age and health habits. Other sleep measures, including sleep fragmentation and self-reported sleep time, obtained via sleep diaries and actigraphy turned out not to be strong predictors for cold susceptibility [30]. Taken together, these studies showed that sleep duration and sleep efficiency are strong predictors for cold susceptibility. 

### 2.4. Severe Acute Respiratory Syndrome Coronavirus-2 (SARS-CoV-2)

Currently, we are in the midst of a pandemic. The virus responsible for this pandemic belongs to the family of coronaviruses and is called SARS-CoV-2. This particular virus causes the disease COVID-19. Worldwide, many people have been infected with the virus and develop the disease every day. The rapid spread of the virus is due to its highly infectious nature. SARS-CoV-2 enters and infects the respiratory tract of humans, thereby causing symptoms that look like that of the common cold. Sneezing, coughing, and shortness of breath are symptoms that could be both due to the common cold or COVID-19. Sudden loss of taste and smell, however, is a symptom very specific to COVID-19. In addition, the disease can also cause pneumonia and eventually respiratory failure, making it more fatal than the common cold. There are over 5.3 million people worldwide that have died because of the virus so far [31]. 

As in the case of the common cold, sleep could be seen as an important component in the protection against COVID-19 by maintaining a functional immune response. There is a dearth of data, however, on the connection between sleep and COVID-19. One study by Kim et al. (2021), for example, studied sleep as a risk factor for COVID-19 among healthcare workers (HCWs) from different countries. HCWs that work in the front-line are especially at a high risk of a COVID-19 infection. It is important to know, particularly in these people, whether sleep has an effect on the risk for COVID-19 infection. In a case-control study, participants were asked to fill out a web-based questionnaire on their sleeping behavior. Results show that a 1-h longer sleep duration at night decreased the odds of a COVID-19 infection by 12% (*p* = 0.003). People who had multiple sleep problems had an 88% greater risk for COVID-19 infection. Napping during the daytime, on the other hand, had a 6% higher chance of COVID-19, but this differed per country, making this result less reliable [32]. The results by Kim et al. (2021) are strengthened by three recently published studies showing an increased change for COVID-19 infection in various shift-working populations [33,34,35].

As previously mentioned, circadian rhythms and sleep are important for immune system homeostasis. A deregulated circadian rhythm could therefore induce a proinflammatory state, making a person more susceptible to COVID-19. HCWs at the front-line are especially at risk of developing circadian rhythm problems due to changes in daily routine and an irregular sleep–wake schedule. To conclude, it is expected that both improved sleep duration and sleep efficiency may reduce the spread and severity of COVID-19, induced by the SARS-CoV-2 virus [36]. 

## 3. Effect of Circadian Rhythms and Sleep on Vaccination Success

We have thus far described the impact of sleep on immune function, and in particular susceptibility to viral infection. It is important to realize that most of the studies within that area studied the effects of sleep on isolated immune parameters. In reality, an effective immune response is more complex and relies on the interaction between multiple immune cells and mediators. Therefore, it is critical to also include studies on the interactions that occur during an immune response. For this, vaccination turns out to be a suitable experimental model, since it resembles infection and can be administered at any given time point in healthy humans. There is growing evidence suggesting that sleep has a critical role in antibody responses after vaccination [13]. 

Proinflammatory cytokine production is increased by experimentally induced sleep deprivation and brief habitual sleep. Prolonged wakefulness increases the number of circulating neutrophils, NK cells, monocytes, and B cells, while recovery sleep decreases them, implying that sleep plays a role in controlling leukocyte trafficking [13,37].

### 3.1. Mechanisms of Vaccine-Induced Immunological MEMORY Formation

Vaccine-induced immunity is mediated by a complex interaction of innate, humoral, and cell-mediated immunity. Vaccines are the most cost-effective of all other life-saving medical interventions, with an estimated 2.5 million lives saved annually. The immunological response to vaccination varies from person to person, both in terms of quantity and quality [38]. 

Vaccines ensure immunological memory. It is thought that sleep supports this immunological memory formation, making vaccination more efficient. Understanding the mechanisms underlying this memory formation might help in understanding the role of sleep in immunological memory. When looking at the memory formation of the CNS, three phases can be distinguished. First, there is the encoding phase, in which relevant information needs to be sensed by our body. In the case of the immune system, this means that antigen-presenting cells (APCs) recognize the foreign antigen. These cells take up the pathogen upon entry and present it to the rest of the body, thereby eliciting an immune response. Second, the consolidation phase ensures the transformation of the relevant information from short-term storage to long-term storage. In the CNS, these storage places are defined as different brain regions. In the immune system, the memory T and B cells are seen as the long-term storage. In secondary lymphatic tissues, information is conveyed from APCs to T cells. T cells that have been activated proliferate and produce effector and memory cells. In addition, B cells are activated and start producing antibodies. Third and last is the recall phase. This is the retrieval of the stored memory, in which memory T and B cells are activated upon re-encounter of the antigen. Sleep appears to support the consolidation phase of immunological memory. Sleep reduces the number of APCs and T lymphocytes in the circulation, causing them to be redirected to the lymph nodes. This increases the likelihood of both cell types encountering each other, thereby transferring information from the APCs to the T cells. In addition, as mentioned in the above, sleep favors a proinflammatory cytokine production, thereby further supporting the interaction between APCs and T cells. For this supportive role of immunological formation, it seems that deep sleep, or slow-wave sleep (SWS), is the most relevant sleep stage [39]. Existing literature supports the concept that SWS promotes and coordinates T cell and APC migration to the lymph nodes as well as communication between the two by producing an immune-supportive hormonal constellation. So far, it only seems that SWS and the association with the unique endocrine constellation are correlational. Nevertheless, a recent study has indicated that boosting slow oscillation activity during sleep in healthy people boosts the hormonal milieu during SWS, lowering the number of circulating T and B cells. This indicates that SWS actively induces an immune-supportive hormonal constellation. Subsequently, this improves the adaptive immune response and ensures a stronger immunological memory [13]. 

Few studies have been done so far on the impact of sleep on antibody responses after vaccination with different types of vaccines. These will be discussed in more detail in the following paragraphs.

### 3.2. Influenza Vaccines

The very first human laboratory study on the impact of sleep on vaccination and its immune response was performed with a vaccine against influenza. For 4 days prevaccination and 2 days postvaccination, participants were restricted to 4 h of sleep per night or either the usual 7.5–8 h per night. During 10 days after the vaccination, influenza-specific antibody titers were measured. It turned out that people who slept 7.5–8 h had more than double the amount of antibody titers compared to the group with restricted sleep [40]. A recent field study on the influenza vaccine found similar results. Healthy young adults had to fill out sleep diaries for 13 consecutive days to assess sleep duration, sleep efficiency, and subjective sleep quality. On day 3, an influenza vaccine was administered. It was found that shorter sleep duration, mainly the two nights before vaccination, resulted in a lower antibody titer 1 and 4 months later. In contrast, sleep efficiency and subjective sleep quality had no influence on the antibody response [41]. In addition, Taylor and colleagues (2017) revealed that poor global sleep quality, measured by the Pittsburgh Sleep Quality Index, predicted poor influenza vaccination response across both insomniacs and noninsomniacs [42].

Another study looked at the effects of a regular 24-h sleep–wake cycle and a 24-h period of continuous wakefulness on the antibody response to the H1N1 (swine flu) vaccination in healthy participants. The antibody production was then followed over time, up to 52 days postvaccination. Researchers found that sleep-deprived men, but not women, showed reduced levels of antibodies five days after the vaccination compared to the sleep group. At later time points, no difference remained in antibody levels between groups. This study shows that sleep has an influence very early in the immune response to a viral antigen [43]. 

### 3.3. Hepatitis Vaccines

Other studies on the influence of sleep on immunological memory formation have looked at vaccines against hepatitis. Lange and colleagues (2011) examined healthy men who were vaccinated three times against hepatitis A. This vaccine is a three-dose series with vaccinations at weeks 0, 8, and 16. The participants were then either assigned to a sleep or wake group for the following night. Sleep was recorded via polysomnography, including EEG, and hormone levels were measured throughout the entire night through sampling blood (via a forearm catheter). In addition, the T cell and B cell (antibody) responses were monitored up to a year after vaccination. Researchers found that antigen-specific T cells were doubled when the participants slept for 7.5 h the night after the vaccination compared to the group who had to stay awake. This finding occurred within 24 h postvaccination, indicating that sleep has an effect early in the immune response, thus most likely on the interaction between APCs and T cells in the lymphatic tissues. The increased number of T cells was accompanied by a greater fraction of proinflammatory cytokine-producing cells, favoring cellular aspects of adaptive immune responses. In addition, SWS stimulated the release of immunosupportive hormones such as growth hormone and prolactin. In contrast, the release of the immunosuppressive hormone cortisol was lowered. The interaction between APCs and T cells and the response to vaccination is under the influence of growth hormones and prolactin [44]. An earlier study of Lange and colleagues (2003) also studied the effects of sleep on the antibody response after hepatitis A vaccination. The night after vaccination, one group had regular sleep whereas the other group stayed awake. The group that had regular sleep showed an antibody titer almost two-fold higher compared to the other group. This was found after 4 weeks of vaccination. It is, however, important to note that no short-term effects were found in this study [45].

Another study vaccinated healthy participants with the hepatitis B vaccine. The researchers wanted to explore if sleep length, sleep efficiency, and (subjective) sleep quality, as measured in the natural environment, could be used to predict the amplitude of antibody responses to a novel antigen. The participants received the standard three-dose vaccination series and sleep measures were assessed using actigraphy and electronic sleep diaries. Antibody titers were collected prior to vaccinations 2 and 3 in order to study the primary and secondary antibody responses. In addition, 6 months after the final immunization the clinical protection status (anti-hepatitis B surface antigen immunoglobulin G ≥ 10 mIU/mL) was assessed. A shorter sleep duration (6 h or less) was associated with a lower secondary antibody response. In addition, the shorter sleep duration decreased the likelihood of being clinically protected after the final immunization. The latter was also confirmed via the self-reported sleep duration using electronic sleep diaries [46]. Contrary to the studies of Lange and colleagues, both short- and long-term effects of sleep on immune responsiveness after vaccination were found. 

### 3.4. Circadian Rhythms and Vaccination Success 

Another important factor to take into account when looking at vaccination efficacy is the circadian rhythm. Our immune system exhibits circadian rhythmicity and therefore the timing of vaccination could also play a key role in the success of vaccination [47]. For instance, a study on hepatitis A and influenza vaccines found higher antibody titers when people were vaccinated in the morning compared to the afternoon. The higher antibody titer was almost two times higher and the effects were still seen 4 weeks after vaccination, an effect only seen in men [48]. More recently, Long et al., 2016 conducted a study showing that morning vaccination significantly increased viral specific antibody responses compared with afternoon vaccination [49]. 

To our knowledge, the vaccination response in people with circadian disruption, i.e., shift workers, has only been investigated very recently. Riuz et al., 2021 examined the immune response on the meningococcal C meningitis vaccine in shift workers and day workers and found that both had a lower total sleep time and that circadian rhythm disruptions were associated with reduced humoral responses after vaccination. They reported that shift workers had a lower SWS and REM duration, associated with higher cytokine levels and a weaker specific leukocyte-mediated immune response to vaccination [50]. 

### 3.5. COVID-19 Vaccines

Both cross-sectional studies and studies using experimentally induced sleep restrictions have found a link between disrupted sleep and circadian rhythms and reduced antibody responses. Thus, shorter sleep duration leads to impairments in cell-mediated immunity across a variety of vaccines. This is a problem for the current COVID-19 pandemic, since, during these times, sleep is often disturbed. A recent study showed that people either slept less or longer than before the COVID-19 pandemic started [51]. During the COVID-19 outbreak in China, data from all the residents on their sleep behavior were collected and showed that more than 20% of the people met the criteria for clinical insomnia. In addition, those people also reported to spend more than 1 h awake during the night. Moreover, it was found that people who are at greater risk for SARS-CoV-2, including healthcare workers, had more severe insomnia [32]. This has a negative effect on both susceptibility to infection and vaccination success [52]. Especially with the current vaccination strategy, it is of great importance that people sleep for 7 h or more every night to ensure a higher vaccination success. Sleep and circadian-based interventions could help optimize healthy sleep and circadian alignment prior to vaccination. 

These limited human studies available provide convincing evidence that circadian alignment and efficient sleep supports vaccination outcomes, but more research is warranted. In 2021, a study was initiated by Lammers-van der Holst and colleagues to investigate the association between sleep and circadian misalignment in relation to immune response to COVID-19 vaccination [53], addressing the pressing need to gain more knowledge on this matter [14,15].

## 4. Discussion

**Figure 1:**
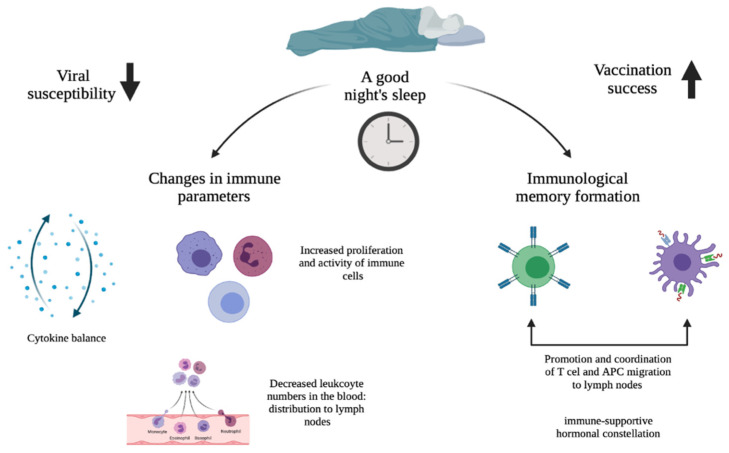
Schematic overview of how sleep and the circadian rhythm potentially affect viral susceptibility and vaccination success: a good night’s sleep on the one hand leads to a more optimal day–night balance in cellular immunity and cytokine production, resulting in reduced viral susceptibility (left downward arrow); on the other hand, it would lead to an improved response to vaccination through a better immunological response to the vaccine antigens (right upward arrow).

The effects of sleep and circadian rhythms on immune function, i.e., susceptibility to viral infections, and vaccination success are topics that go hand-in-hand and therefore deserve investigation. Figure 1 highlights the main paths by which sleep and circadian rhythms affect viral susceptibility and vaccination success, as described in detail in this review. It would appear that sleep (deprivation) has an effect on multiple immune parameters. For this, the circadian rhythm plays a critical role. Since sleep and the circadian rhythm are very much intertwined, this means that immunological activities affected by insufficient sleep also influence immunological activities that are affected by circadian disruption. In the case of susceptibility to viral infection, it was found that sleep and circadian disruptions alter cytokine production, which results in a chronic imbalance in immune homeostasis in favor of a proinflammatory state. This constant proinflammatory environment increases the susceptibility to viral infections by breaking down the immune tolerance. For the influence on vaccination success, it turned out that sleep duration and time of day were associated with the magnitude of the antibody response after vaccination. Immunological response to vaccination ensures immunological memory and sleep supports this memory formation. In particular, it was found that SWS promotes and coordinates T cell and APC migration to the lymph nodes as well as communication between the two by producing an immune-supportive hormonal constellation. Subsequently, this improves the adaptive immune response and ensures a stronger immunological memory. 

The studies reviewed show some inconsistencies in their results, due in part to certain limitations. First, it is important to note that the majority of the studies done on the effects of sleep and circadian rhythms on immune function investigated individual immunological markers/immune parameters. In reality, an effective immune response is much more complicated, requiring the interaction of multiple immune cells and mediators of both the innate and the adaptive immune system. This is critical to take into account when interpreting the results. Relevant findings will therefore always have to be studied in the context of an ongoing immune response. Vaccination has proven to be a good experimental model for this situation, since it resembles infection and can be administered to healthy people at any time. However, studying the effects of sleep and circadian rhythms on vaccination success showed discrepancies. For instance, every study on the subject has shown a different persistence of the sleep effect, i.e., a different response in antibody titer during a certain time period postvaccination. On the one hand, studies showed increased levels of antibody titers up to 4 or 6 months after vaccination when participants slept more than 7 h per night. On the other hand, the study on the H1N1 vaccination reported no differences in antibody levels between the sleep and wake group 52 days postvaccination. These differences in the persistence of the sleep effect could be due to the variations in study design and the type of virus that was used. However, on the whole, these studies do provide evidence that sleep duration, around the time of vaccination, is an important factor in boosting the host’s immune response and immunological memory. Another explanation for discrepancy in results could be that the effect size of sleep deprivation on adaptive immune responses is antigen-specific. Research into the mechanisms connecting circadian and sleep disruption to adaptive immune functions is still sparse and more research is highly needed. 

Another limitation in some of these studies is the way of measuring sleep duration in the participants. Self-reporting is a subjective way of measuring sleep and is subject to recall bias. People tend to overestimate their sleep duration and underestimate the time/minutes spent awake during the night. Next to measuring sleep duration, differences in the type of sleep manipulation used in studies exist. Studies have either used total sleep deprivation, partial sleep deprivation (i.e., a few hours less sleep per night), or REM sleep deprivation. In addition, the total duration of the sleep manipulation differed per study. This was divided into two categories: acute effect of sleep and effect of prolonged wakefulness. It is important to note that it is difficult to compare these studies due to the substantial methodological differences and that any inconsistencies found are partly attributable to these differences. 

### 4.1. Implications for the COVID-19 Pandemic 

All these findings offer an important perspective for the COVID-19 pandemic that is currently ongoing. The few studies that have already been done on the SARS-CoV-2 virus are in line with the rest of the literature and state that sleep and circadian rhythms are associated with susceptibility to infection and most likely vaccination success. Based on the literature found, it is therefore crucial to sleep for 7 h or more every night to decrease the risk of contracting COVID-19. Nonetheless, this is easier said than done. Especially HCWs, who are at greater risk, were found to have severe insomnia. Due to night shifts, an irregular sleep–wake schedule, and changes in daily routine, circadian rhythm problems are easily developed. This has a negative effect on both susceptibility to infection and vaccination success, rendering the issue of high importance. A good night of sleep prior to vaccination and the night after vaccination can increase the antibody response, boosting immunization efficacy against the SARS-CoV-2 virus. By introducing sleep and circadian-based interventions, vaccination immunity will likely be enhanced, leading to an improvement in global health. Research to date remains sparse, indicating the importance of further research within this area. 

### 4.2. Recommendations 

As mentioned in the limitations, the studies on vaccination success all showed differences in the persistence of the sleep effect. It has been shown that obtaining sufficient sleep exerts a positive effect on the antibody response after vaccination by increasing the antibody titer, but it remains unclear as to why there are differences in the duration of this effect. It is therefore recommended that more research is done on this topic to gain a better understanding. For this, it is important that studies are more comparable by being transparent in their designs so that variations in outcome are less likely due to these differences in study design. 

When looking at study design, it is particularly important to measure sleep duration, since this seems to be the only sleep measure associated with antibody response so far. To judge the extent to which sleep duration has an influence on the antibody response, study designs could possibly divide participants into multiple groups based on individual sleep need, comparing sufficient sleepers to sleep-deprived people. It is of note that the effects of sleep on immune function predominantly use experimental manipulations of sleep, while there is a dearth of data on immune response in chronically sleep-deprived or sleep-disordered people.

Clinical recommendations would consist of, first, ensuring adequate sleep and optimal circadian rhythmicity, adhering to sleep hygiene measures or aspects of cognitive behavioral therapy for insomnia—CBTI; second, it would seem, based on findings for hepatitis A and influenza vaccines, that vaccination is particularly effective when administered in the morning. A third recommendation would be the use of melatonin. While melatonin may be useful to align circadian rhythms, it is also known for its anti-inflammatory, immunomodulatory, and antioxidant mechanisms [54]. Two recent clinical trials have noted promising results, but also point to the need for further investigation [55,56]. Since melatonin has a high safety profile, a low cost, and is widely available, this may represent an interesting compound to further study in the light of viral infections and vaccination. 

## 5. Conclusions

Taken together, the results in these studies show that sleep, primarily sleep duration, has a critical role in the functioning of the immune system. Obtaining sufficient sleep, ~7 h or more every night, will improve immune function, thereby making people less susceptible to viral infection and increasing the magnitude of the antibody response after vaccination. With a large percentage of the population experiencing numerous forms of circadian disruptions ranging from minor weekend delayed sleep patterns to severe rotating night-shift work, many individuals are potentially at risk of being relatively immune-compromised. This review highlights the importance of recommending sufficient sleep and circadian alignment to optimize our immune functions. 

There are still gaps in the current knowledge that need to be filled by doing more research. In the future, it could be interesting to study differences between individuals that exist in regard to their habitual sleep need and chronotype. For example, this research could focus on the differences between morning and evening types of people or the social jetlag phenomenon in which one’s biological rhythm and social life misalign. Finally, a better understanding of the sleep–immune relationship across various time dimensions is critical to develop interventions that ensure an optimal immune function and sleep health. 

## Data Availability

Not applicable.
